# Scalability of carbon-nanotube-based thin film transistors for flexible electronic devices manufactured using an all roll-to-roll gravure printing system

**DOI:** 10.1038/srep14459

**Published:** 2015-09-28

**Authors:** Hyunmo Koo, Wookyu Lee, Younchang Choi, Junfeng Sun, Jina Bak, Jinsoo Noh, Vivek Subramanian, Yasuo Azuma, Yutaka Majima, Gyoujin Cho

**Affiliations:** 1 Department of Printed Electronics Engineering, Sunchon National University, Sunchon 540-742, Korea; 2 Regional Innovation Center for Printed Electronics, Sunchon National University, Sunchon 540-742, Korea; 3 Department of Electrical Engineering and Computer Sciences, University of California, Berkeley CA 9472033, USA; 4 Materials and Structure Laboratory, Tokyo Institute of Technology, Yokohama 226-8503, Japan

## Abstract

To demonstrate that roll-to-roll (R2R) gravure printing is a suitable advanced manufacturing method for flexible thin film transistor (TFT)-based electronic circuits, three different nanomaterial-based inks (silver nanoparticles, BaTiO_3_ nanoparticles and single-walled carbon nanotubes (SWNTs)) were selected and optimized to enable the realization of fully printed SWNT-based TFTs (SWNT-TFTs) on 150-m-long rolls of 0.25-m-wide poly(ethylene terephthalate) (PET). SWNT-TFTs with 5 different channel lengths, namely, 30, 80, 130, 180, and 230 μm, were fabricated using a printing speed of 8 m/min. These SWNT-TFTs were characterized, and the obtained electrical parameters were related to major mechanical factors such as web tension, registration accuracy, impression roll pressure and printing speed to determine whether these mechanical factors were the sources of the observed device-to-device variations. By utilizing the electrical parameters from the SWNT-TFTs, a Monte Carlo simulation for a 1-bit adder circuit, as a reference, was conducted to demonstrate that functional circuits with reasonable complexity can indeed be manufactured using R2R gravure printing. The simulation results suggest that circuits with complexity, similar to the full adder circuit, can be printed with a 76% circuit yield if threshold voltage (*V*_*th*_) variations of less than 30% can be maintained.

Over the years, the vision of realizing extremely inexpensive thin film transistor (TFT) integrated electronic circuits through an all roll-to-roll (R2R) printing process has been pursued as an advanced and sustainable manufacturing method for rollable and disposable electronic devices. To date, however, no realistic demonstrations of such fabrication processes have been achieved in a scalable R2R manner. Here, for the first time, a R2R gravure printing system, including relevant inks and substrates, is employed to successfully demonstrate fully printed thin film transistor (TFT)-based rollable and disposable electronic devices.

To enable the realistic fabrication of electronic devices using R2R gravure printing, the attainable range of threshold voltage (*V*_*th*_) variation in R2R gravure-printed TFTs must first be determined to enable the design and implementation of circuits with appropriate threshold voltage levels^1^,^2^. The *V*_*th*_ variation is directly related to the various trapped charges (N_tr_(t)) and the gate oxide capacitance (C_o_) in printed TFTs (ΔV_th_(t) = eN_tr_(t)/C_o_)^3^ and to the printed physical dimensions, such as the thickness, surface roughness and edge waviness of printed patterns. Therefore, to employ the R2R gravure system for manufacturing printed TFT-based flexible and disposable circuits, the *V*_*th*_ variation in R2R gravure-printed TFTs should first be defined and controlled to enable a suitable level of device integration. Because currently available commercial R2R gravure machines cannot provide registration accuracies of greater than ±10 μm, the designed TFT dimensions must be selected to be compatible with these constraints. In other words, when larger channel lengths and wider gate electrodes are employed, higher yields of R2R gravure-printed TFTs can be expected. Furthermore, the R2R gravure-printed gate electrodes used in this work utilize silver nanoparticle-based ink, which has a surface roughness in the range of 30 to 100 nm^4^. Therefore, the R2R gravure-printed dielectric layers should be sufficiently thick (>1 μm) to provide planarization on the rough printed gate electrodes and thus avoid device short circuits through the gate dielectric. Given this use of thicker dielectric layers, employing materials with a high dielectric constant (>10) is necessary for achieving sufficient electrostatic control of the channel.

In general, the rough surface (rms >5 nm) and thick layer (>1 μm) of printed dielectric layers (low capacitance) are difficult to avoid or substitute if a full R2R gravure printing process is employed for fabricating TFT-based electronic devices on a plastic roll. To compensate for the rough surface and low capacitance of the R2R gravure-printed dielectric layer, robust semiconducting materials with high mobility are highly desirable. In this system, single-walled carbon nanotubes (SWNTs) would be a good semiconducting layer because SWNTs will form a random network structure after printing (likely more tolerant to disorder than, for example, a film formed using crystal organic semiconductors), and a very high carrier mobility in the range of 10,000 cm^2^/Vs is obtained when pure semiconducting SWNTs are used^5^,^6^. For this reason, SWNT-based TFTs (SWNT-TFTs) have been extensively proposed as a semiconducting ink for realizing inexpensive, flexible and partially printed electronic devices, such as flexible TFT backplanes^7^,^8^,^9^, logic circuits^10^, and RF circuits^11^. In fact, a SWNT channel with high mobility would compensate for the performance-degrading effects of surface roughness of printed dielectric layers with low capacitance and long channel lengths^12^,^13^. However, to practically realize this objective, such devices should be fully R2R printable using a high-throughput printing process, such as R2R gravure, on plastic foils with a minimal variation in the device performance. To fully employ R2R gravure in printing SWNT-TFTs, the SWNT-based ink should have a good viscosity to homogeneously cover the rotating gravure cylinder for transferring an even amount of SWNTs onto the moving web. Moreover, the ink should be dried in less than 7.5 s to provide a minimum web transfer speed of 8 m/min given a reasonable drying zone length. Similar constraints apply for the conductive ink used to print gate, source and drain electrodes, and for the dielectric ink used to print dielectric layers. Additionally, the overlay printing registration accuracy must be controlled within ±10% of the width of the gate electrodes in both the machine and transverse directions. To satisfy these conditions, while providing a sheet resistance of less than 1 Ω/sq for printed electrodes and a capacitance of at least 6 nF/cm^2^ for printed dielectric layers, commercially available silver-nanoparticle-based inks and BaTiO_3_-nanoparticle-based ink were selected because they exhibited good electrical properties for printing TFTs with a web transfer speed of up to 12 m/min^14^.

In this work, we used R2R gravure printing in conjunction with a SWNT-based semiconducting ink, a silver-nanoparticle-based ink, and a BaTiO_3_-nanoparticle-based dielectric ink to print SWNT-TFTs with five channel lengths (30, 80, 130, 180, and 230 μm) on a 150-m-long roll of PET with a web transfer speed of 8 m/min (Fig. 1). Based on the ±20 μm registration accuracy of the R2R gravure printing system, a constant gate width of 350 μm was used while varying the channel length between 30 and 230 μm. The selected width of the gate electrode and channel length is very important for obtaining a high device yield because any excessive misalignment between the drain-source electrode and the gate electrode will cause device failure. Therefore, the width of the gate electrodes was designed to be sufficiently wide to account for the aforementioned overlay printing registration limits. Finally, the scalability of the R2R gravure system for printing SWNT TFT-based electronic circuits was demonstrated. A 1-bit adder circuit integrating 53 SWNT-TFTs was used as a reference circuit and simulated using Monte Carlo simulations with three different ranges of *V*_*th*_ variation (10, 20, and 30%) to refine the optimal device dimension for manufacturing the 1-bit adder circuit using the R2R gravure printing system.

## Results

**Figure 1:**
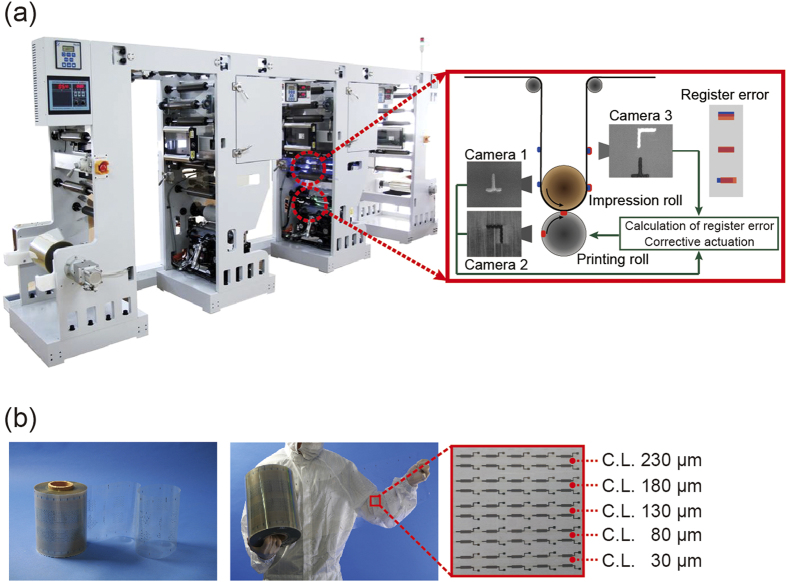
R2R gravure to fully print SWNT-TFTs. (**a**) R2R gravure with video-camera-based servomechanism to control the overlay printing registration accuracy of the machine and transverse directions to ±20 μm along with the descriptive lay out for controlling the overlay registration accuracy of R2R gravure up to ±20 μm using a three CCD camera system. (**b**) Image of R2R gravure-printed SWNT-TFTs with 5 different channel lengths (30 to 230 μm) on 150-m-long PET web.

By utilizing the three aforementioned nanomaterial-based inks (silver-nanoparticle-based ink, BaTiO_3_-nanoparticle-based ink and SWNT-based ink) and R2R gravure printing, the attainable overlay printing registration accuracy should first be characterized prior to defining the printed TFT dimensions. In this work, an R2R gravure printer, with two printing units (Fig. 1a), was designed to test the three different nanomaterial-based inks by feeding a 0.25-m-wide poly(ethyleneterephtalate) (PET) web. The web tension and pressure of the impression roller were maintained with accuracies of ±0.3 kg_f_ and ±0.38 psi, respectively. The control systems for the web tension and impression roller pressure were established based on an ideal gravure cylinder roll with a perfect circumference. However, because realistic manufactured gravure cylinders typically have an imperfect circumference, the registration markers on the same gravure cylinder roll generally vary within a maximum range of ±20 μm (Fig. S1 in the Supplementary Information). Therefore, we specially designed the system with three CCD cameras to control the registration accuracy at ±20 μm in both the machine and transverse directions (Fig. 1a) even with the imperfect circumference of the gravure cylinder roll. The first camera detected the printed registration markers after passing through the first printing unit. The second camera detected the registration markers on the gravure cylinder at the second printing unit. The obtained images of both markers are used to define the position error values resulting from the mismatch between the two detected images based on the machine and transverse directions. The errors are corrected by decreasing or increasing the rotation speed of the gravure cylinder and by precisely moving the gravure cylinder in the lateral direction. Finally, the matching of two markers was monitored by the third camera, which provided feedback to the control program. Based on this servomechanism, a registration accuracy of ±20 μm at a web transfer speed of 8 m/min was attained. In addition, the unit actively responded to the expansion of the plastic web (PET ~ −0.6 mm/m) while passing through the heating chamber (150 °C).

**Figure 2:**
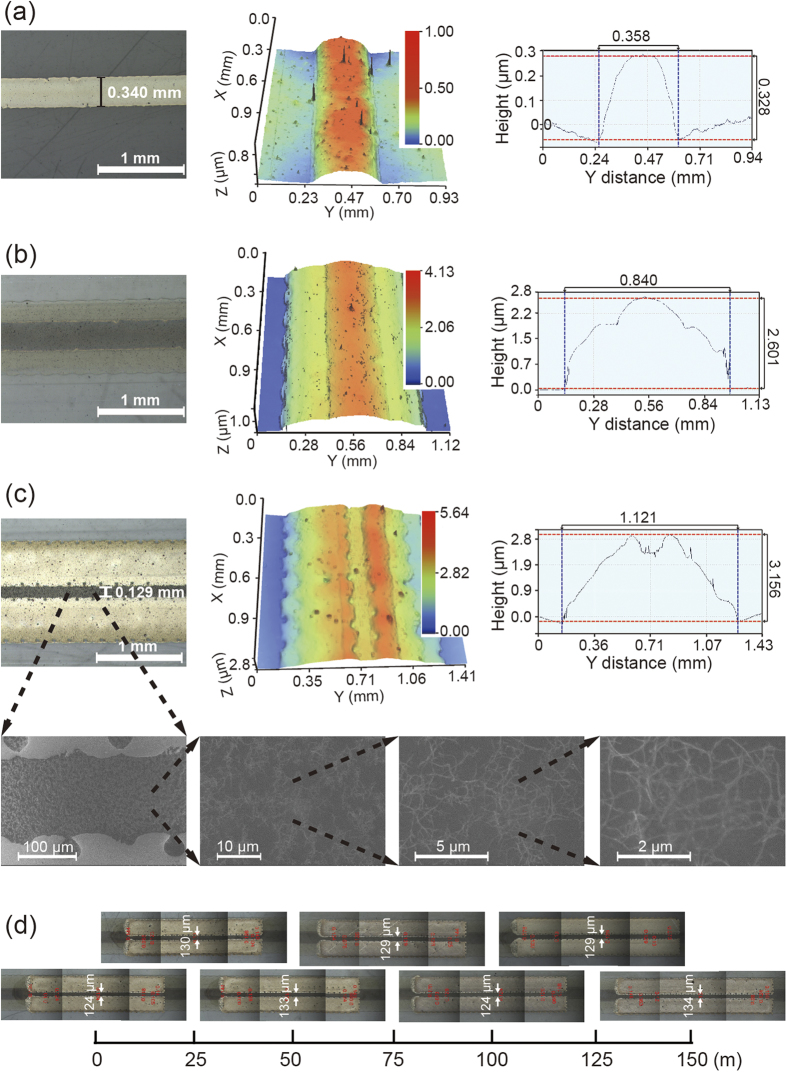
Step by step image analysis of R2R gravure-printed layers for SWNT-TFTs. SEM and surface morphology images with cross-sectional analysis of R2R gravure-printed (**a**) gate electrodes, (**b**) dielectric layers, and (**c**) drain-source electrodes with a network structure of SWNT layers along 150 m of PET web. Image (**d**) shows homogeneously R2R gravure-printed drain-source electrodes with the channel length of 130 μm on the 150-m-long PET roll as an example.

To fully print SWNT-TFTs on PET foils using R2R gravure, as shown in Fig. 1b, we first needed to determine the optimal printing conditions for printing gate electrodes and dielectric layers with low surface roughness using commercially available silver-nanoparticle-based and BaTiO_3_-nanoparticle-based inks. These inks were further adjusted to meet the required rheological characteristics (viscosities of 500 and 100 cP, respectively, and surface tensions of 48 and 31.6 mN/m, respectively). The average surface roughnesses obtained for the gate electrodes and dielectric layers were 31 nm and 51 nm (rms), respectively (Fig. 2). After drying the entire length (150 m) at 150 °C for 15 s, the R2R gravure-printed gate electrodes showed thicknesses of 328 ±32 nm (Fig. 2a) with a typical resistance of 2.5 mΩ/sq/mil. In this R2R gravure process (Fig. 1a), the 15 s drying step for gate electrodes was achieved by passing the web through two of 1-m-long heating zone at a web speed of 8 m/min. The dielectric layers were subsequently printed on the printed gate electrodes at the second printing unit. The resulting dielectric layers were observed to possess a thickness of 2.6 μm (Fig. 2b) and a capacitance of 7 nF/cm^2^ without any pin holes. Printing the dielectric layers is the step that has the greatest influence on the performance of printed TFTs because a height difference of 328 nm and wetting differences between the printed gate and PET substrate surfaces will often generate pin holes and inhomogeneous printed dielectric layers. An 800-μm-width dielectric layer was empirically selected to overcome these issues and to avoid the height and wetting differences between the printed-gate electrodes and bare substrate^13^ (refer to Fig. S2 in the Supplementary Information).

SWNTs have never been R2R gravure printed in a roll-based process; rather, they have only been printed using a roll-to-plate gravure^1^,^15^,^16^ because a rotating gravure cylinder cannot pick up the SWNT-based ink from the ink reservoir. Therefore, in this work, a novel spraying method was employed to spray the SWNT-based ink (0.016 wt% SWNTs with a viscosity of 24 cP) on the rotating gravure cylinder. Although the viscosity of SWNTs can be increased to an appropriate level (>100 cP)^17^,^18^ for R2R gravure, the electrical performance of SWNT-TFTs with high viscosities will be extremely poor due to the polymer binders typically used as a viscosity-increasing agent. After printing the SWNT layer with R2R gravure, as shown in the insets of Fig. 2c, drain-source electrodes were gravure-printed with channel lengths of 30, 80, 130, 180, and 230 μm. Figure 2c,d also show that, for printed channel lengths of 130 μm, the variation in the channel lengths is in the range of ±5 μm, and the edge waviness of the printed drain-source electrodes is almost constant (approximately 10.5 μm). Comparison of the two samples with extreme edge waviness (3.0 μm and 9.4 μm) of drain-source electrodes for each channel length revealed that the waviness of the R2R gravure-printed drain-source electrodes did not severely influence the electrical parameters of the SWNT-TFTs because of the relatively large channel lengths^19^ (refer to Fig. S3 in the Supplementary Information).

**Figure 3:**
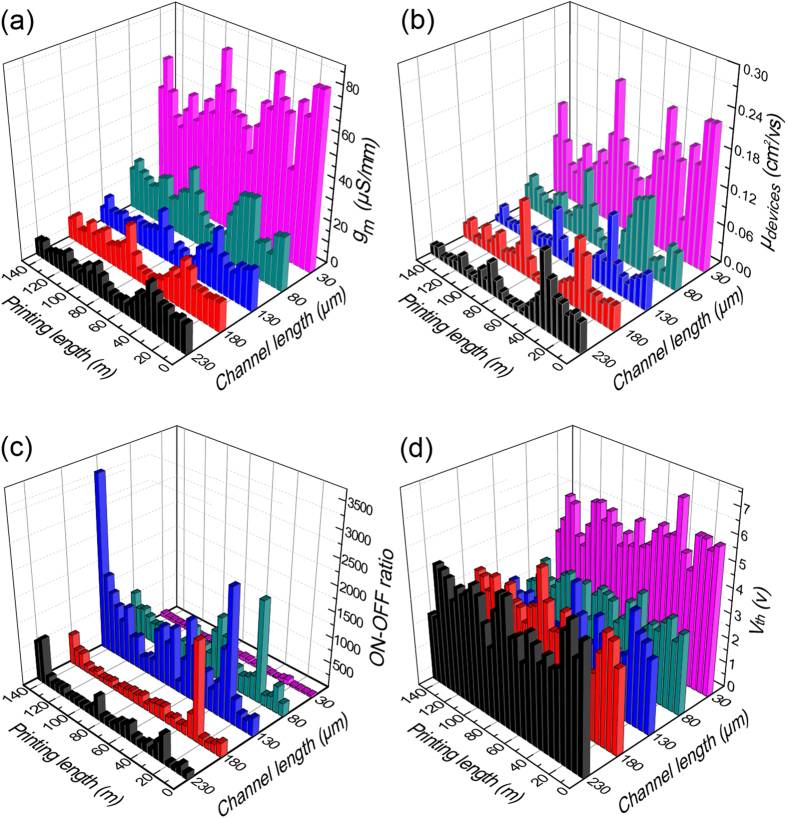
Statistical characterization of R2R gravure-printed SWNT-TFTs on 150 m of PET web. Distribution of average values of (**a**) transconductance (*g*_*m*_), (**b**) carrier mobility (*μ*_devices_), (**c**) ON-OFF current ratio, and (**d**) threshold voltage (*V*_th_) of SWNT-TFTs with five different channel lengths at every 5 m along 150 m of PET web.

**Figure 4:**
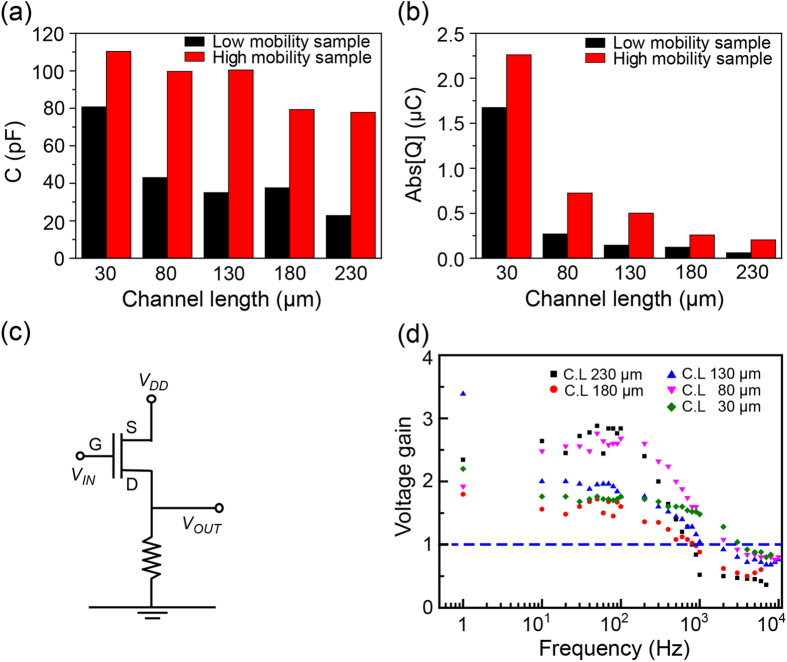
Characterization of printed SWNT-channel and cutoff frequency of R2R gravure-printed SWNT-TFTs. (**a**) Capacitances at accumulation and (**b**) channel charges of printed SWNT-TFTs with randomly selected samples under different channel lengths (30, 80, 130, 180, and 230 μm) on a 150 m length of PET web. (**c**) Layout of inverter circuit with load resistance to test the cutoff frequency of printed SWNT-TFTs. (**d**) Observed cutoff frequency of fully R2R gravure-printed SWNT-TFTs with high mobility by constructing an inverter with a resistor.

Statistical characterization of the R2R gravure-printed SWNT-TFTs was performed by measuring the electrical characteristics of every fifth TFT. The typical output and transfer characteristics of the SWNT-TFTs for each channel length are shown in Fig. S4 in the Supplementary Information. The mobility was determined using the following equation: *μ*_device_ = (*L/V*_D_*C*_ox_*W)(dI*_d_*/dV*_g_) = *(L/V*_D_*C*_ox_*)(g*_*m*_*/W*) with a gate oxide capacitance per unit area (*C*_ox_) of 7 ± 0.8 nF/cm^2^ on average^20^. This parallel-plate capacitance is an overestimate because of the non-homogeneous coverage of SWNTs on the active area^21^,^22^. Therefore, the extracted mobility values in this work were consistently lower than the real values. However, these values are still a reasonable assumption for circuit speed estimation because errors in the estimation of the effective mobility and driving capacitance largely cancel out. The variations in normalized transconductances (*g*_*m*_*/W*), field-effect mobility at *V*_ds_ = −20 V, ON-OFF current ratio and *V*_*th*_ for each channel length along the 150 m of PET web are shown in Fig. 3. As it is often observed for SWNT-based TFTs^23^,^24^ in this work, the transconductance and the mobility reached maximum average values of 81.3 μS/mm and 0.23 cm^2^/Vs, respectively, at the shortest channel length (30 μm) (Fig. 3a,b). Because of metallic SWNTs in the SWNT ink and because of the length of the SWNTs, the ON-OFF current ratio decreased at these shorter channel lengths (Fig. 3c). As shown in Fig. 3, the observed average transconductance and mobility for all channel lengths in the PET web fluctuated together at the positions of 35, 85, 110, 125, and 140 m because these two values are proportionally interrelated (*μ*_devices_ = 2*Lg*_*m*_/*WC*_ox_). The variations in *V*_th_ for all printed SWNT-TFTs are shown in Fig. 3d. The *V*_*th*_ variations of the printed SWNT-TFTs of all channel lengths were in the range of 19 to 34% in this R2R system. The lowest variation (19%) was observed at the channel length of 30 μm whereas the highest variation 34% was observed at the channel length of 130 μm. Because the variations in channel lengths and gate dielectric capacitances of the TFTs were not very large through the entire length of 150 m, the other possible reasons for the observed notable fluctuations in the electrical properties of the printed SWNT-TFTs are from the misalignment of the drain-source and gate electrodes and from the unequal network density of the printed SWNTs. Therefore, we compared the relationship between the degree of misalignment and transconductance using three extreme samples for high and low *g*_*m*_ for channel lengths of 30, 80, 130, 180, and 230 μm. The effect of misalignment on *g*_*m*_ was not obvious because long channel lengths and wide gate electrodes were used, which were sufficient for the rated registration accuracy of ±20 μm of the R2R gravure (refer to Table S1 and Fig. S5 in the Supplementary Information). However, during the R2R gravure printing process, the amount of SWNT ink transferred from the gravure cylinder to the printed dielectric layers slightly varied along the 150 m of PET web due to the low viscosity of the SWNT ink and the surface roughness of the printed dielectric layers. The quantitative network density of SWNTs is difficult to statistically measure directly using high-resolution scanning electron microscopy because the area is large. Therefore, the accumulation capacitance of the printed SWNT channels was employed to indirectly compare the network density of the printed SWNTs^25^ in each SWNT-TFT. Measuring the *C*_*accumulation*_ of SWNT channels from the results of capacitance-voltage (*C-V*) measurements (Fig. S6 in the Supplementary Information) at the gate and source electrodes for randomly selected SWNT-TFTs with low and high mobilities with five different channel lengths will provide relative information about the network density of printed SWNT. The relative network densities of the SWNTs in these R2R gravure-printed SWNT-TFTs can be indirectly compared by estimating the channel charges (Q) using the following equation^26^: Q = C_channel accumulated_/WL (*V*_*gs*_ − *V*_*th*_) (Fig. 4a,b). The higher mobility samples had a higher channel charge due to the higher network density of printed SWNTs. Based on these results, we can hypothesize that the variation in *V*_th_ would be more vulnerable to the network density of printed SWNTs than to the physical parameters of the printed SWNT-TFTs or any other printing factors, such as the overlay printing registration accuracy, edge waviness and surface roughness of printed layers, in this R2R gravure system. Furthermore, because SWNT ink contains polymer binder, this binder would act as fixed charge sources. By considering the obtained values of the ON-OFF current ratio, *g*_*m*_, *μ*_devices_ and *V*_*th*_ variation from the SWNT-TFTs along the 150 m of PET, the SWNT-TFTs with channel length of 130 and 180 μm will be the most appropriate TFT dimensions for constructing SWNT TFT-based functional devices using this R2R gravure system. This hypothesis was further validated by Monte Carlo simulations, as shown in the discussion section.

To evaluate the operating frequency of R2R gravure-printed SWNT-TFTs, we constructed an inverter using a driver TFT, SWNT-TFT and a commercially available resistor (Fig. 4c). We experimentally estimated the attainable operating frequency of the TFT with the highest and average values of the mobility under five different channel lengths by simply measuring the maximum inverting frequency at a gain of one (Fig. 4d). Moreover, the theoretical cutoff frequency was calculated using the equation *f*_T_ = *g*_*m*_/2*π(C*_GS_ + *C*_GD_), where *C*_GS_ and *C*_GD_ are the parasitic capacitances generated by drain-source overlap to gate. At the maximum mobility of the channel length of 30 μm, the calculated cutoff frequency was 581 kHz. However, the experimentally observed value was 3 kHz; this is likely due to parasitic capacitances and dispersive effects not accounted for in the DC characterization of the devices.

## Discussion

**Figure 5:**
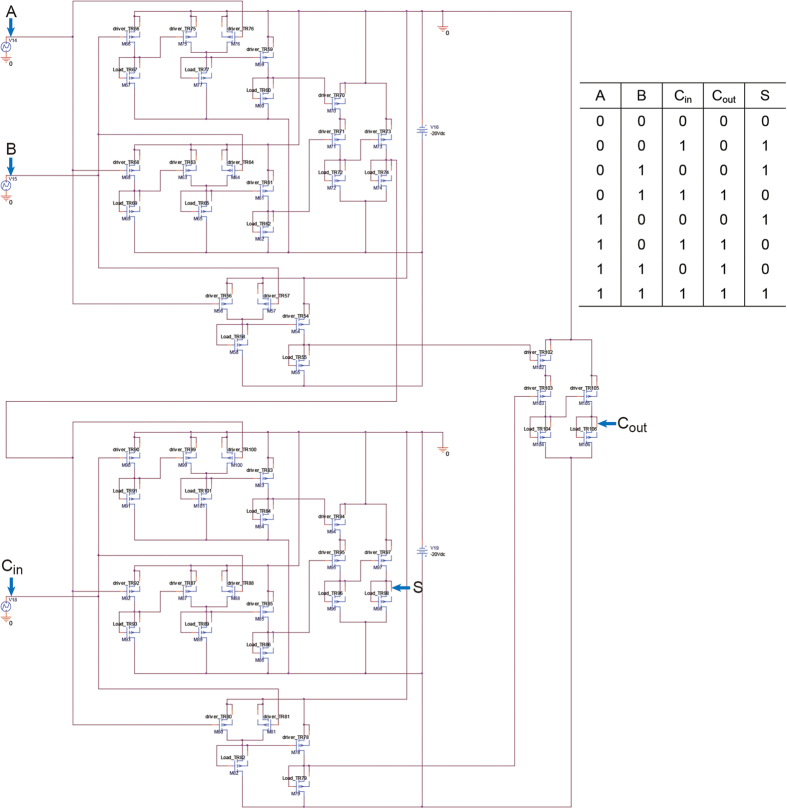
A circuit layout of 1-bit adder. Circuit and truth table of 1-bit adder circuit using the printed 53 SWNT-TFTs.

**Figure 6:**
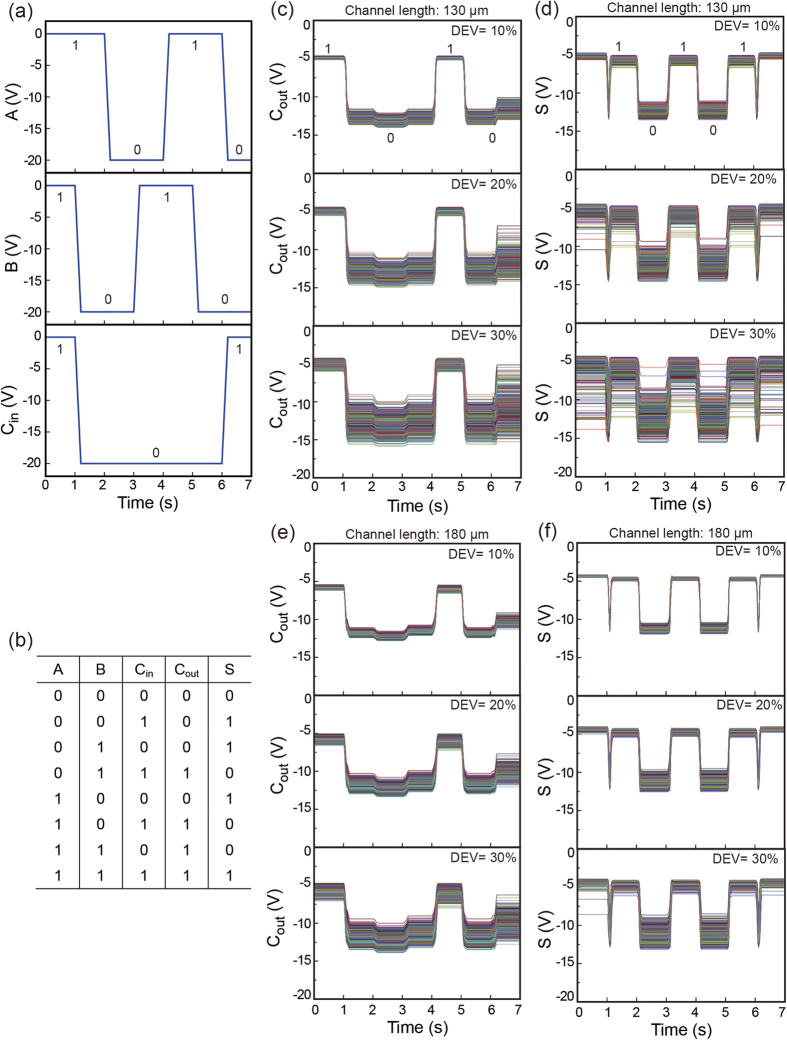
Monte Carlo simulations of 1-bit adder circuit. (**a**) Results of Monte Carlo simulations on the 1-bit adder circuit with input signal and (**b**) truth table using the extracted electrical parameters from R2R gravure-printed SWNT-TFTs with channel lengths of 130 μm (**c,d**) and 180 μm (**e,f**).

As mentioned in the introduction, the operation of printed logic circuits is strongly impacted by variations in the component TFTs within the circuits in question. In other words, as *V*_*th*_ variation in the logic circuit increases, only very simple logic functions can be operated. This is a major reason why fully R2R-printed flexible or rollable logic devices have not yet been demonstrated. However, the ultimate goal and innovative applicability of printed electronics is to manufacture disposable devices with an extremely low cost, which is only achievable using a high-throughput printing method such as R2R gravure. Therefore, the practical demonstration of fully R2R gravure-printed logic devices is the highest priority in the era of printed electronics. The scalability of the R2R gravure system for manufacturing flexible and rollable devices was indirectly proven through the Monte Carlo simulations using the extracted electrical parameters from forward *IV* measurements in R2R gravure-printed SWNT-TFTs with channel lengths of 30 to 230 μm. A 1-bit adder circuit with 53 SWNT-TFTs (Fig. 5) was used for the simulation. By employing a *V*_*th*_ variation of 30% in the fully R2R gravure-printed SWNT-TFT, the full adder or less than 53 SWNT TFT-based electronic devices can be manufactured with a circuit yield of 76% for the 130 μm channel length case. However, the circuit yield was improved up to 100% by decreasing the *V*_*th*_ variation to 10% (Table 1 and Fig. 6). As indicated by the simulation results, the dimensional matching between printed SWNT-TFTs and the registration accuracy of this R2R gravure system is critical for obtaining practical device yields. In this R2R gravure system, a channel length of 130 μm and gate width of 350 μm were the best device dimensions for manufacturing SWNT TFT-based electronic devices with less than 53 SWNT-TFTs. Based on currently available R2R gravure systems, large size and low functionality electronic devices obtained by integrating a number of SWNT-TFTs would be achievable by simply maintaining the current variation range of *V*_*th*_. Furthermore, because the same range and patterns of electrical parameters (*g*_*m*_, *μ*_devices_, ON-OFF current ratio, and *V*_th_ ) of R2R gravure-printed SWNT-TFTs were repeated on the 150 m of PET web using the same printing system (refer to Fig. S7 in the Supplementary Information), this R2R gravure system has been demonstrated to be a practical method for fabricating SWNT-TFTs and will become an advanced manufacturing method for producing rollable and disposable electronic devices.

In conclusion, a R2R gravure system, including inks and substrate, was demonstrated to be a scalable printing method for manufacturing more than 150,000 rollable and disposable electronic devices. This was achieved by printing SWNT-TFTs along 150 m of PET web with a minimum channel length of 30 μm and with 100% yield in only 60 min by controlling the web tension with an accuracy of ±0.3 kg_f_, an impression roller pressure with an accuracy of ±0.38 psi, and an overlay printing registration accuracy of ±20 μm. The attained maximum carrier mobility and transconductance were 0.30 cm^2^/Vs and 93.24 μS/mm, respectively, at a channel length of 30 μm. The maximum ON-OFF current ratio of 5200 was attained at the channel length of 130 μm. Monte Carlo simulations of R2R gravure-printed SWNT-TFTs demonstrated that this R2R gravure system is sufficient for mass producing flexible and disposable low functionality electronic devices with moderate complexity. Based on the indirect relationship between the variation in *V*_*th*_ and the density of printed SWNT along 150 m of web, the range of *V*_*th*_ variation was minimized by controlling the transfer of SWNT ink during R2R gravure printing. The circuit yield was improved to more than 90% at a channel length of 130 μm using this R2R gravure system. Therefore, this R2R gravure system using nanomaterial-based electronic inks would be used to manufacture actual devices with simple functions for a range of low-cost electronic applications.

## Methods

### Printing system and inks

The R2R gravure system (Fig. 1a) was manufactured by iPen Co., Korea. Two color printing units were utilized to fabricate SWNT-TFTs on a roll of PET film (thickness of 100 μm, SKC, Korea). Commercially available silver-nanoparticle-based ink (PG-007, Paru Co., Korea), BaTiO_3_-nanoparticle-based ink (PD-100, Paru Co., Korea) and SWNT-based ink (PR-040, Paru Co., Korea) were purchased and further formulated to adjust their wettability and viscosity to print the gate and drain-source electrodes, dielectric layer, and active layer, respectively.

The details of the ink formulation for fine tuning of each ink to meet the wettability and viscosity requirements and the printing conditions are summarized in Table 2.

Because the R2R gravure system has only two printing units, the gate electrodes and dielectric layers were first continuously R2R printed. After completely printing the gate electrodes and dielectric layers, the PET web was rewound. Subsequently, the active layers were printed at the second printing unit using SWNT ink. After printing the SWNT layers, the PET web was re-wound and then the printed drain-source electrodes were printed at the second printing unit. The total printing time to print 150,000 SWNT-TFTs on 150 m of PET web was approximately 60 min using the R2R gravure system with 2 printing units.

### Measurements

The surface tensions and viscosities of the inks were measured using a DCAT21 (Dataphysics Co., Germany) and a SV-10 Vibro viscometer (AND Co., Japan), respectively. An LCR meter (E4980A, Agilent, USA) and semiconductor characterization system (KEITHLEY 4200, USA) were used to measure *C-V* between the gate and source electrodes and to characterize the printed SWNT-TFTs on every 5 m of PET web along 150 m. The morphology of the printed SWNT-TFTs was measured using a surface profiler (NV-220, Nanosystem, Korea). All measurements were obtained under ambient conditions.

## Additional Information

**How to cite this article**: Koo, H. *et al.* Scalability of carbon-nanotube-based thin film transistors for flexible electronic devices manufactured using an all roll-to-roll gravure printing system. *Sci. Rep.* **5**, 14459; doi: 10.1038/srep14459 (2015).

## Supplementary Material

Supplementary Information

## Figures and Tables

**Table 1:** A summary of Monte Carlo simulations.

**Channel length(μm)**	**Device yields (%)**		
	**In DEV 10%**	**In DEV 20%**	**In DEV 30%**
30	the circuit cannot operate based on the Pspice simulation		
80	the circuit cannot operate based on the Pspice simulation		
130	100	91.6	76.4
180	85.2	65.8	56.4
230	the circuit cannot operate based on the Pspice simulation		

Lists of device yields from Montel Carlo simulation on 1-bit adder circuits based on SWNT-TFTs (53 SWNT-TFTs) with channel lengths of 30 to 230 μm with a different range of *V*_*th*_ variation.

**Table 2:** Brief summary of electronic inks used in the R2R gravure printing.

	**Ag ink for gate electrodes**	**BaTiO_3_ ink for dielectric layer**	**SWNT ink for active layer**	**Ag ink for source-drain electrodes**
Surface tension (mN/m) at 20 °C	48	31.6	25.5	36
Viscosity (cP)	500	100	24	1500
Additives for tuning viscosity of ink	Dipropylene glycol methyl ether	2-(2-Ethoxyethoxy)ethyl acetate	Diethylene glycol monobutyl ether	Dipropylene glycol
Methods of fine tuning ink for optimizing printability	Mixture of Ag ink (PG-007, Paru Co.) (120 g) and additive (2 g) was mechanically stirred for 1 h	Mixture of dielectric ink (PD-100, Paru Co.) (82.5 g) and additive (15 g) was sonicated for 8 h	Mixture of SWNT ink (PR-040, Paru Co.) (36.5 g) and additive (1.5 g) was sonicated for 2 h at 0 °C	Mixture of Ag ink (PG-007, Paru Co.) (120 g) and additive (8 g) was mechanically stirred for 1 h
Printing conditions				
	Printing speed (m/min)	Printing pressure (kg_f_)	Tension(kg_f_)	Drying temperature (°C)
Gate electrodes	8	6	5	150
Dielectric layers	8	6	5	100
Active layers	8	6	5	150
Drain-source electrodes	6	6	5	150

Lists of additives for tuning a surface tension and a viscosity for printing gate electrodes, dielectric layers, active layers and drain-source electrodes using the R2R gravure.
