# Supplementary material for: Scalability of carbon-nanotube-based thin film transistors for flexible electronic devices manufactured using an all roll-to-roll gravure printing system
Source: Sci Rep. 2015 Sep 28;5:14459. doi: 10.1038/srep14459 (PMC4585984; doi:10.1038/srep14459)
Supplement: Supplementary Information [file srep14459-s1.pdf]

Scalability of carbon-nanotube-based thin film transistor for a flexible electronic device through an all roll-to-roll gravure printing system

Hyunmo Koo[1], Wookyu Lee[2], Younchang Choi[1], Junfeng Sun[1], Jina Bak[2], Jinsoo Noh[1], Vivek Subramanian[3], Yasuo Azuma[4], Yutaka Majima[4], and Gyoujin Cho[1,2*]

[1]*Department of Printed Electronics Engineering, Sunchon National University, Sunchon 540-742, Korea*

[2]*Regional Innovation Center for Printed Electronics, Sunchon National University, Sunchon 540-742, Korea*

[3]*Department of Electrical Engineering and Computer Sciences, University of California, Berkeley CA 9472033, USA*

[4]*Materials and Structure Laboratory, Tokyo Institute of Technology, Yokohama 226-8503, Japan*

Supplementary Information

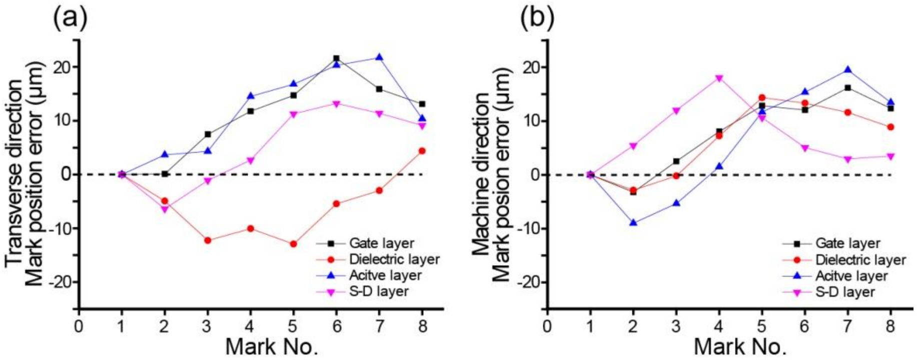

Figure S1. Registration markers on a gravure cylinder roll show a mismatch within the range of ±20 μm.

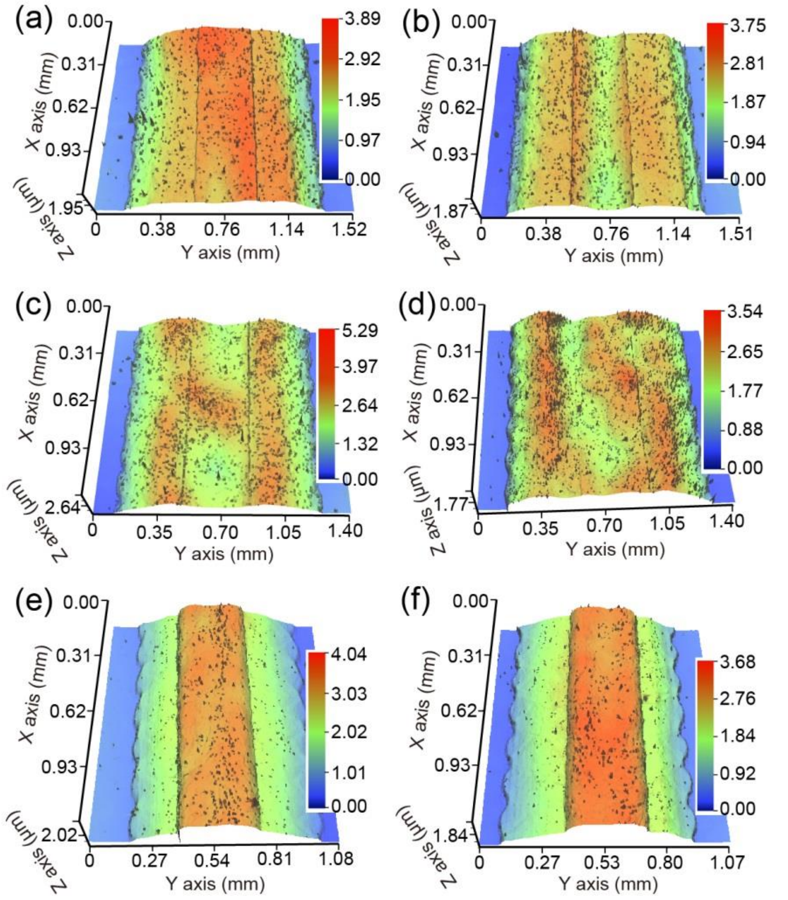

Figure S2. Morphology of R2R gravure-printed dielectric layer with line widths of 1.1 mm (a)–(d) and 0.8 mm (e)–(f), using various viscosities of the dielectric ink: (a) 125, (b) 160, (c) 200, (d) 250, (e) 100, and (f) 120 cP.

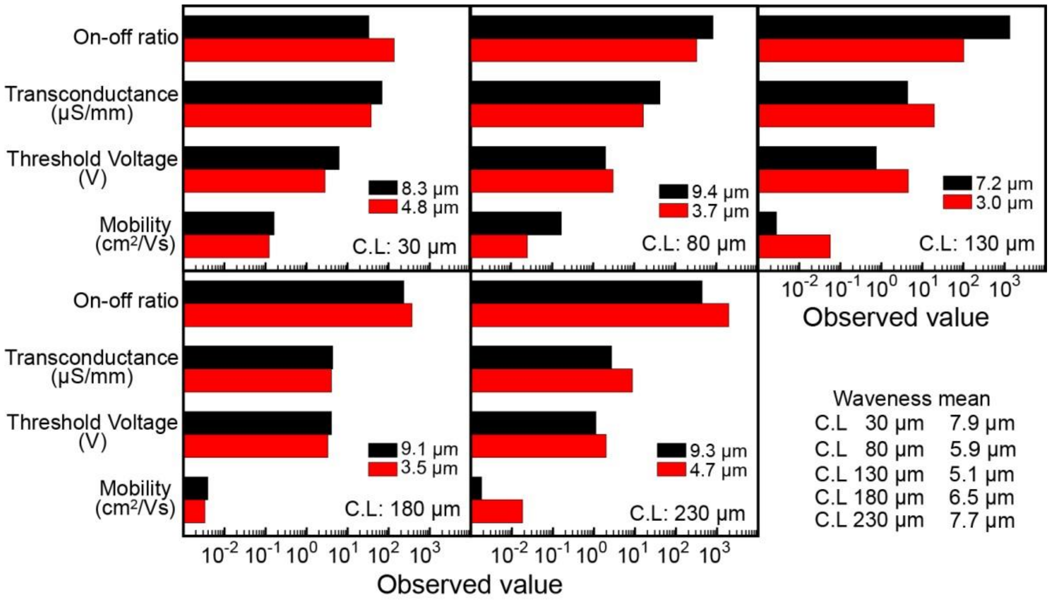

Figure S3. Variations in key electrical characteristics from two extreme samples of source-drain edge waviness of printed SWNT-TFTs with channel lengths of 30, 80, 130, 180, and 230 µm.

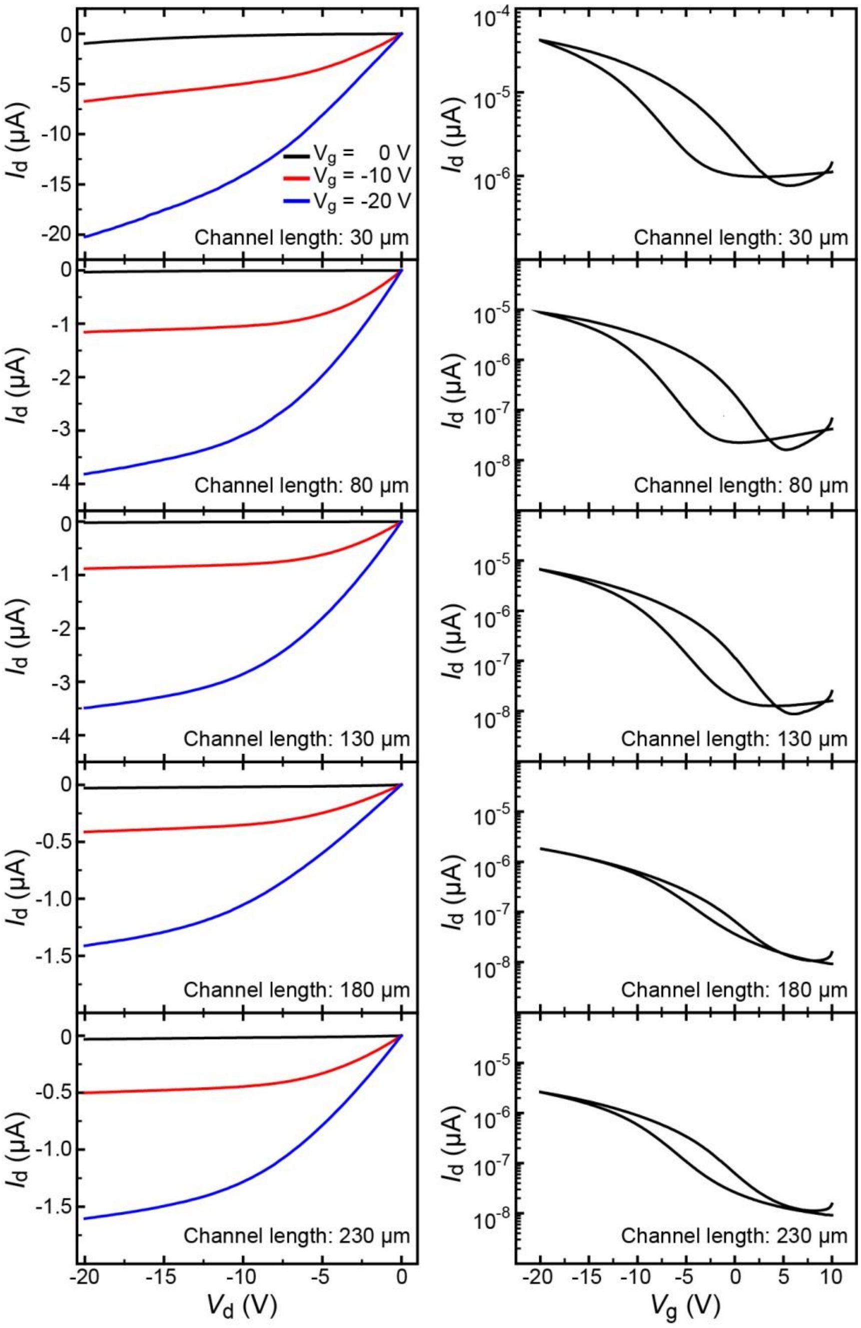

Figure S4. Typical output and transfer characteristics of printed SWNT-TFTs with channel lengths of 30, 80, 130, 180, and 230 μm.

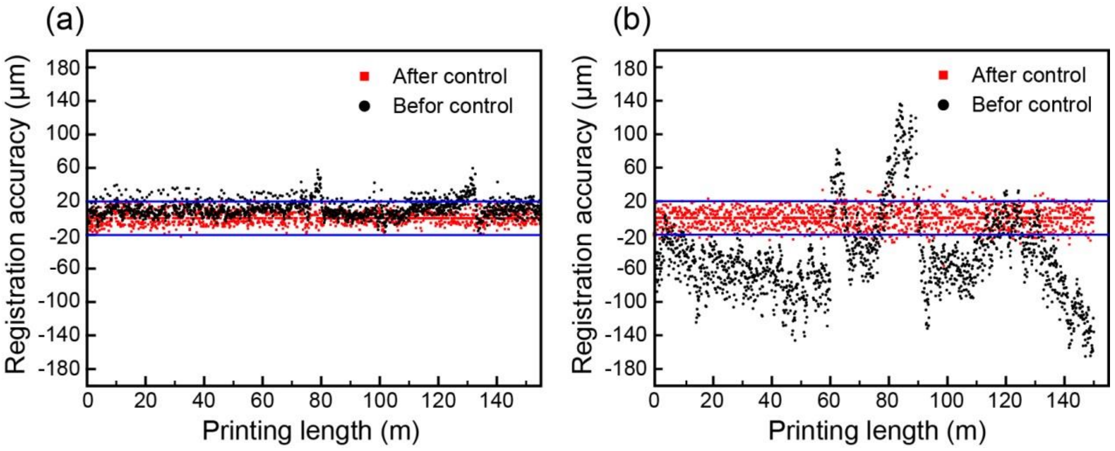

Figure S5. Attained values of registration accuracy of R2R gravure with machine (a) and transverse (b) directions at the printing speed of 8 m/min.

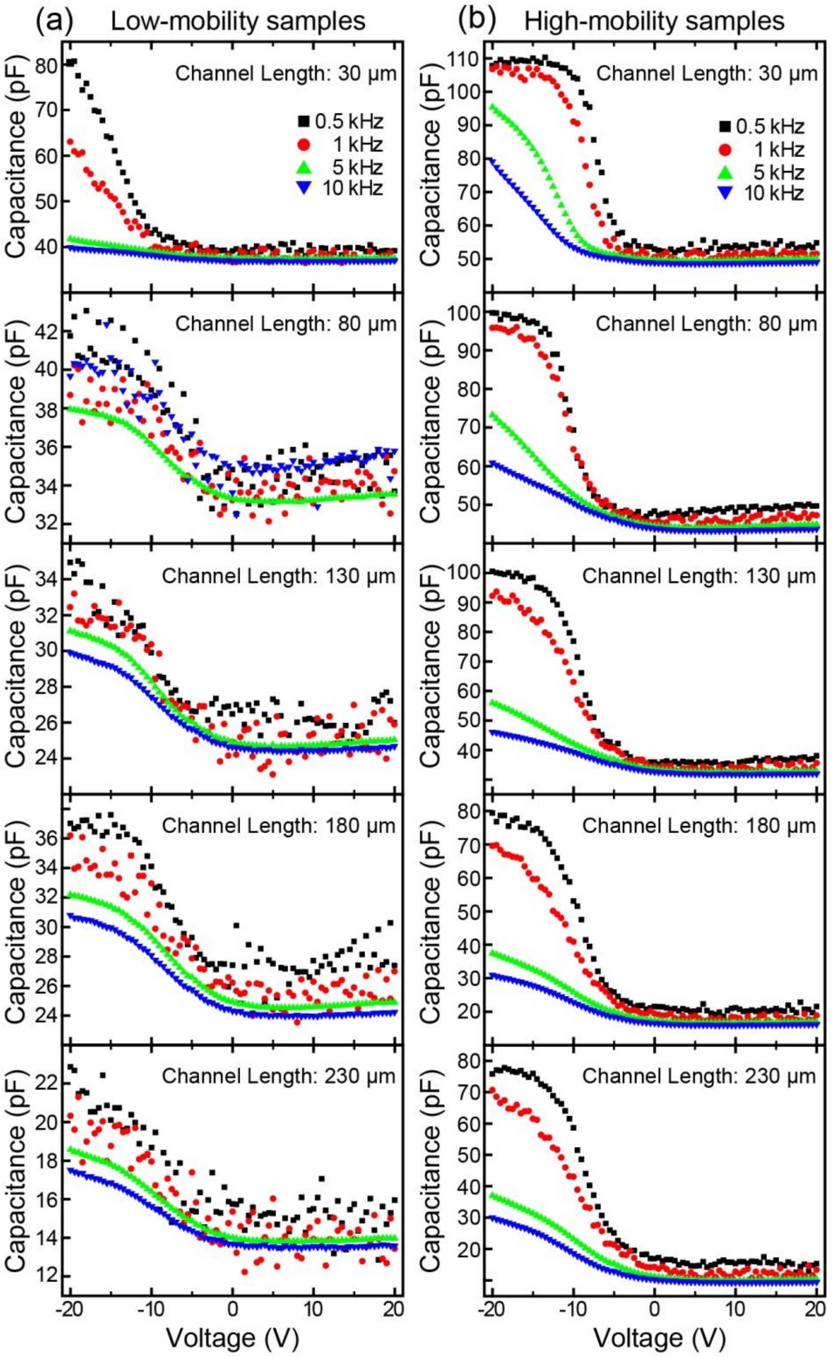

Figure S6. Results of *C*-*V* measurements between gate and source electrodes for both (a) low and (b) high mobility of printed SWNT-TFTs with all five different channel lengths.

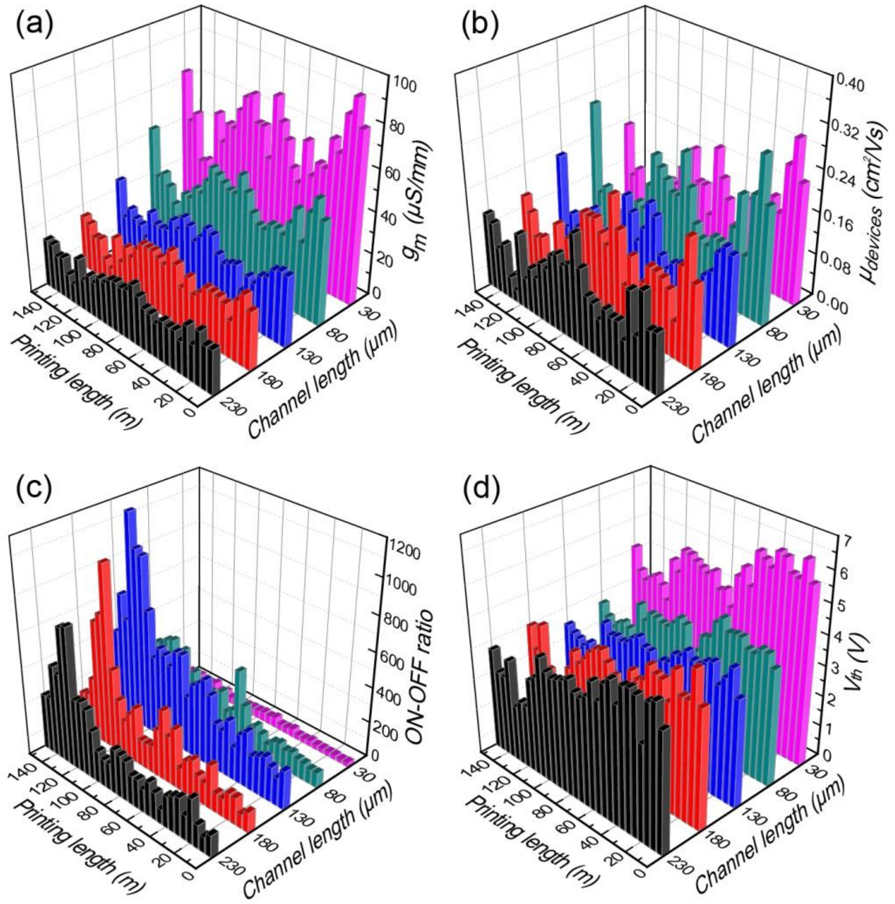

Figure S7. Distribution of average values of (a) transconductance (g_m), (b) carrier mobility ($\mu_{devices}$), (c) ON-OFF current ratio, and (d) threshold voltage (V_{th}) of SWNT-TFTs with five different channel lengths at every 5 m along 150 m of PET web under 2nd printing to observe reliability of R2R gravure system.

Table S1. Relationship between the degree of misalignment of drain-source electrodes and attained transconductance values from printed SWNT-TFTs.

Channel length (µm)	Transconductance (µS/mm)	Superposition area (mm^2) A source & gate	B drain & gate	∣A – B∣ (mm^2)
230	25.54	0.219	0.212	0.007
	44.29	0.248	0.208	0.040
	37.16	0.230	0.174	0.056
	14.66	0.301	0.084	0.217
	8.54	0.213	0.175	0.036
	13.20	0.230	0.163	0.067
180	55.39	0.268	0.296	0.028
	40.32	0.311	0.267	0.044
	38.01	0.319	0.253	0.066
	18.51	0.340	0.253	0.087
	12.05	0.359	0.213	0.128
	14.69	0.414	0.178	0.236
130	53.57	0.418	0.423	0.005
	62.05	0.396	0.419	0.023
	52.12	0.426	0.374	0.052
	13.04	0.393	0.361	0.035
	35.48	0.417	0.394	0.023
	12.16	0.482	0.304	0.178
